# A novel inflammation-based prognostic index for patients with esophageal squamous cell carcinoma: Neutrophil lymphocyte ratio/prealbumin ratio

**DOI:** 10.1097/MD.0000000000014562

**Published:** 2019-02-15

**Authors:** Yajuan Lv, Jiandong Zhang, Zhen Liu, Yuan Tian, Fengjun Liu

**Affiliations:** Department of Radiation Oncology, Shandong Provincial Qianfoshan Hospital affiliated to Shandong University, Jinan, Shandong, PR China.

**Keywords:** esophageal squamous cell carcinoma (EC), neutrophil lymphocyte ratio/prealbumin ratio (NLR/PA), prognosis

## Abstract

NLR/Alb (neutrophil lymphocyte ratio/albumin ratio), is a prognostic index for esophageal cancer has been confirmed. Prealbumin (PA) is more sensitive to malnutrition than albumin. A new prognostic index, named neutrophil lymphocyte ratio/prealbumin ratio (NLR/PA), for predicting the survival time in patients with esophageal squamous cell carcinoma (ESCC) was proposed.

A retrospective study of 315 cases with ESCC was enrolled. The optimal cut-off values were evaluated by ROC curve (the receiver operating characteristics curve). Pearson correlation analyses were used to calculate the correlations among NLR, Alb, NLR/Alb and NLR/PA. The overall survival (OS) was calculated by Kaplan-Meier method. Cox regression analyses were performed to evaluate the prognostic factors.

The optimal cut-off value was 0.01 for NLR/PA according to ROC curve. According to multivariate analyses, TNM stage, NLR, NLR/Alb, NLR/PA were prognostic factors for OS. The AUC area (the area under the receiver operating characteristics curves) of the NLR/PA was higher than the areas of NLR and NLR/Alb for all the patients. The index of NLR/ PA had a higher AUC area than that of the index of NLR or NLR/Alb for patients in stage I-II. But in stage III-IVA, the index of NLR had a higher AUC area than that of the index of NLR/PA or NLR/Alb.

The index of NLR/PA is superior to the index of NLR as a prognostic indicator for patients with early stage (stage I-II) ESCC.

## 1 Introduction

Esophageal cancer (EC) is one of the most prevalent malignant diseases worldwide, and has the sixth mortality rates of any cancer globally.^[1]^ An increasing number of EC are diagnosed worldwide every year. Squamous cell carcinoma (ESCC) is the main pathological type in China. Although there are many therapies for EC, such as surgery, radiotherapy, targeted therapy and immunotherapy, the prognosis for EC remains poor. Surgery remains the first choice for patients with localized EC. And the 5-year survival rate of EC patients treated with surgery is less than 30%.^[2]^ It is very necessary to find an effective prognosis indicator for patients with EC. To date, several tumor markers such as carcinoembryonic antigen (CEA) have been used to predict the prognosis of EC, but the values remain limited. The status of nutrition and inflammation plays an important role in cancer progression and prognosis.^[3–5]^ And a series of inflammatory markers, such as neutrophil lymphocyte ratio (NLR) has been confirmed to predict the prognosis in patients with EC.^[6–7]^ And low serum albumin concentration is also showed to be a predictor of poor prognosis in patients with EC.^[8]^ Recently, a new index named neutrophil lymphocyte ratio/albumin ratio (NLR/Alb) was reported to correlate with prognosis for patients with EC.^[9]^ Prealbumin (PA) has a shorter half-life and is more sensitive to malnutrition than albumin.^[10]^ However, no study so far has reported the prognosis effect of the NLR/PA ratio in patients with ESCC. Therefore, the purpose of this study was to evaluate the prognostic value of preoperative NLR/PA in ESCC patients. It also showed whether the index of NLR/PA had an advantage over than NLR or NLR/Alb.

## 2 Methods

### 2.1 Patients

Between January 2008 and June 2013, a total of 315 patients with ESCC were included in the current retrospective study. This trial was approved by the ethical committee of Qianfoshan Hospital. The last follow-up was June 30, 2018. The eligibility criteria were included:

1. first pathologically diagnosed with ESCC and was no chemotherapy or radiotherapy before surgery;
2. aged from 18 to 75 years old and with KPS ≥ 70;
3. R0 resection;
4. No distant metastasis and no history of another primary cancer or with any liver diseases;
5. no hematologic disorders or inflammatory or autoimmune diseases;
6. no serious medical diseases that may affect survival.

### 2.2 Data collection

Blood samples such as neutrophil count, lymphocyte count, albumin levels, and so on were obtained prior to surgery within 2 weeks. The clinicopathological data such as age, gender were obtained from the patients’ medical records. All patients were staged assessed according to the 8th version of the TNM staging system by AJCC (American Joint Committee on Cancer) and UICC (Union for International Cancer Control) for ESCC.

### 2.3 Definitions of the indexes

The index of NLR was defined as the absolute neutrophil count divided by the absolute lymphocyte count. NLR/Alb was defined as NLR divided by the albumin. NLR/PA was defined as NLR divided by the prealbumin.

### 2.4 Statistical analysis

The optimal cut-off values for NLR, NLR/Alb, and NLR/PA were calculated by ROC curve. Pearson correlation analyses were used to calculate the correlations among the indexes of NLR, Alb, NLR/Alb, and NLR/PA. A *t* test or chi-squared test was used to compare the clinical differences among different subgroups. Kaplan-Meier methods were used to analyze OS. And OS was defined from the date of surgery to death due to any cause. The differences of OS inter-groups were compared using the log-rank test. Prognostic factors were evaluated using univariate and multivariate analyses with the Cox proportional hazard regression model. All the statistical tests were 2 sided, and *P* < .05 was considered statistically significant, and confidence intervals (CI) were calculated at the 95% level. All the data was analyzed using SPSS (version 22.0, IBM). Comparison of AUC area among these indexes using MedCalc and *P* < .05 was considered statistically significant.

## 3 Results

### 3.1 Patient characteristics

There were a total of 315 patients enrolled in this study. The median age was 59 years old, with an age range of 35 to 75 years old. Among all the patients, 56 (17.78%) were women and 259 (82.22%) were men. And the 5-year survival rate of all the enrolled patients was 37.14%. The numbers of patients from staged I to IVA were 67, 96, 113, 39 respectively. And there were 184 patients received adjuvant therapy including radiotherapy or chemotherapy after surgery. According to the AUC curve, the optimal cut-off values for NLR, Alb, PA, NLR/Alb and NLR/PA were 3.18, 45 g/L, 221.4 mg/L, 0.06, and 0.01, respectively. According to the cut-off value of these indexes, all the patients were divided into the high and the low group respectively. No significant differences were found between the low and high groups in terms of TNM stage, age, sex, smoking, drinking, and differentiation (Table 1).

**Table 1:**
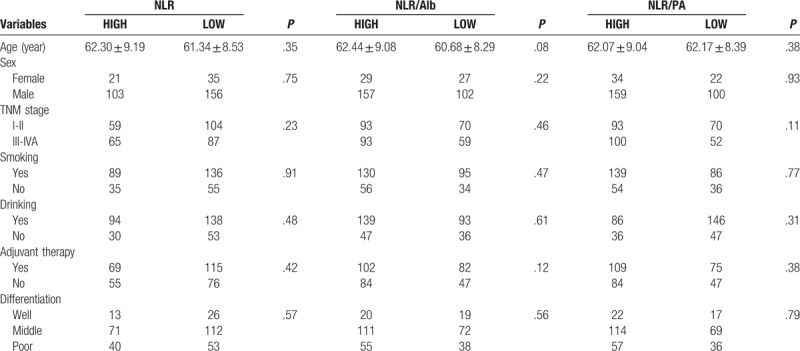
Clinical characteristics in all the patients.

### 3.2 Pearson correlation

**Figure 1:**
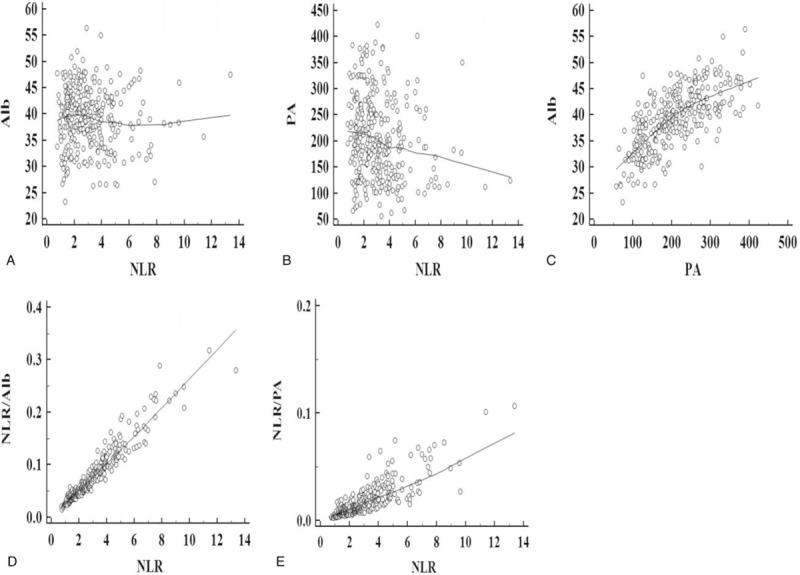
Pearson correlation analysis. Negative correlations between NLR and Alb (r = −0.054, *P* = .341, A), NLR and PA (r = −0.167, *P* = .002, B). Positive correlations between PA and Alb (r = 0.689, *P* < .001, C), NLR and NLR/Alb (r = 0.956, *P* < .001, D), NLR and NLR/PA (r = 0.821, *P* < .001, E).

Pearson correlation analyses revealed that there were negative correlations between NLR and Alb (r = −0.054, *P* = .341, Fig. 1A), NLR and PA (r = −0.167, *P* = .002, Fig. 1B). However, there were positive correlations between PA and Alb (r = 0.689, *P* < .001, Fig. 1C), NLR and NLR/Alb (r = 0.956, *P* < .001, Fig. 1D), NLR and NLR/PA (r = 0.821, *P* < .001, Fig. 1E).

### 3.3 Kaplan-Meier analyses

**Figure 2:**
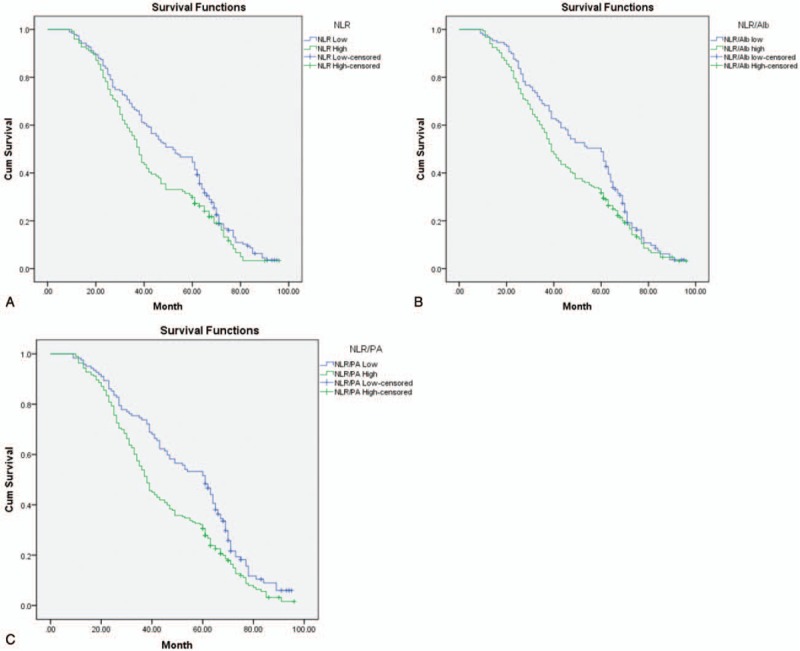
Kaplan-Meier survival curves for OS in 315 patients. (A) NLR; (B) NLR/Alb; (C) NLR/PA.

The OS of patients was significantly longer in the low NLR group compared with that in the high NLR group (*P* = .025, Fig. 2A). And there was a significantly better OS in patients with NLR/PA ≤ 0.01 than patients with NLR/PA > 0.1 (*P* = .001, Fig. 2C). Patients in the NLR/Alb high group had worse OS than patients in the NLR/Alb high group (*P* = .043, Fig. 2B).

### 3.4 Univariate and multivariate analysis

The results were shown in Table 2. Cox univariate analysis indicated that TNM stage, tumor differentiation, NLR, Alb, PA, NLR/PA and NLR/Alb were prognostic factors for OS. However, multivariate analysis revealed that only TNM stage, NLR, NLR/PA and NLR/Alb were significant prognostic factors for OS (*P* < .05).

**Table 2:**
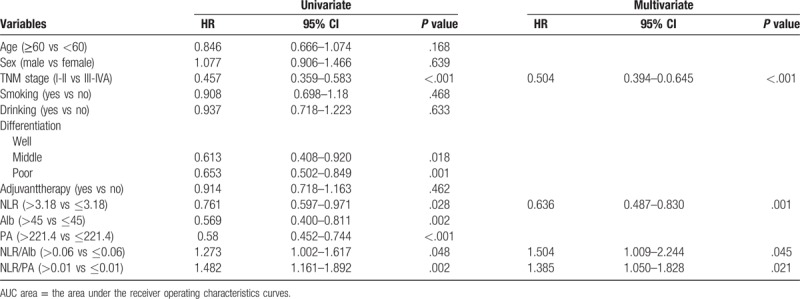
Univariate and Multivariate analyses of OS for all patients.

### 3.5 Comparison of ROC curves of this 3 indexes

**Figure 3:**
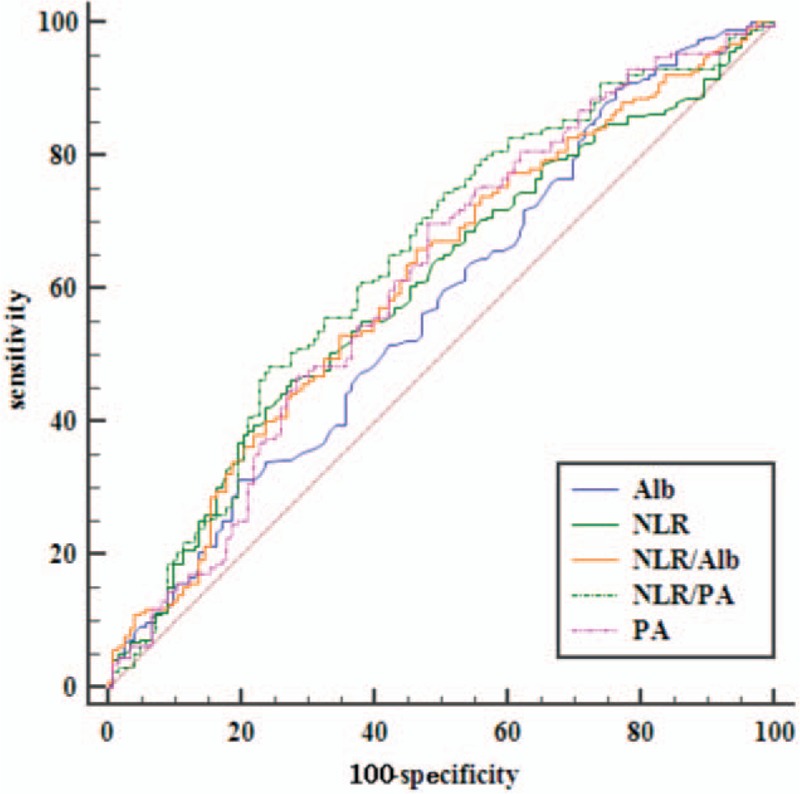
Comparisons of the AUC areas of these index for OS. AUC area = the area under the receiver operating characteristics curves.

**Figure 4:**
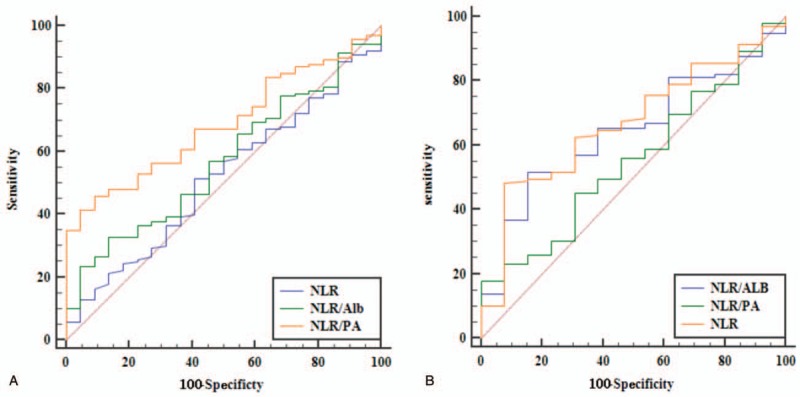
Comparisons of the AUC areas of the 3 index for OS in different stages. (A) stage I-II; (B) stage III-IVA. AUC area = the area under the receiver operating characteristics curves.

The prognostic value of each index was evaluated by comparing the AUC area calculated for the patients, overall survival (Fig. 3). The AUC area was 0.600, 0.575, 0.614, 0.611, and 0.643 for NLR, Alb, PA, NLR/Alb, and NLR/PA, respectively. As shown in Table 3 and Figure 3, the index of NLR/ PA had a higher AUC area than that of the index of NLR or NLR/Alb (*P* = .032). However, there were no statistical differences between the AUC areas of NLR and NLR/Alb (Table 3). And there were also no significant differences in AUC areas of PA when compared with that of Alb or NLR in the overall survival (*P* > .05, Table 3). As shown in Figure 4A, in stage I-II, the index of NLR/ PA (AUC = 0.685) had a higher AUC area than that of the index of NLR (AUC = 0.515, *P* = .037) or NLR/Alb (AUC = 0.568, *P* < .001). But in stage III-IVA (Fig. 4B), the index of NLR (AUC = 0.669) had a higher AUC area than that of the index of NLR/PA (AUC = 0.554, *P* = .023) or NLR/Alb (AUC = 0.643, *P* = .157).

**Table 3:**
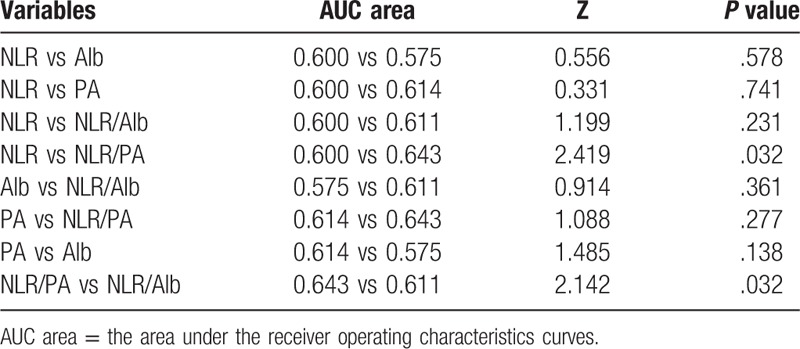
Comparison of AUC area for OS prediction.

## 4 Discussion

Esophageal cancer, as one of the most common malignant tumors in the world, ranks 5th among all malignant tumors in China according to World Cancer Report of WHO in 2014. The incidence of EC in China accounted for about half of the world and the mortality rate is as high as 4.9%.^[9]^ Malnutrition is common in patients with malignant tumor, especially in patients with EC.^[11]^ Preoperative nutritional status of patients with EC has been reported to be closely linked to the OS and the incidence of postoperative complications.^[12]^ In recent years, some inflammatory indexes have provided a new pattern for estimating the prognosis of patients with EC after surgery, such as NLR, C-reactive protein and so on.^[13,14]^ The protein in blood can reflect the nutritional status of the human body.^[15]^ Of the 29 studies reviewed on cancers of the gastrointestinal tumor, all except 3 found higher albumin levels to be associated with better survival in multivariate analysis. And the optimal cut-off value of Alb remains unclear and the reported range of values from 3.5 g/dL to 4.15 g/dL.^[15]^ A study also demonstrated that hypoalbuminemia was an independent prognostic indicators of survival in EC patients.^[16]^ Albumin had close correlations with postoperative complications and prognosis in patients with EC.^[17]^ However, the preoperative serum albumin was not a prognostic index in another study.^[18]^ And this study also showed that the NLR was a prognostic index only in the univariate analysis but not in the multivariate analysis. A meta analysis involving 1633 patients with EC demonstrated that high NLR was significantly associated with poor OS and disease-free survive (DFS).^[13]^ In another meta-analysis, authors demonstrated that elevated NLR was associated with worse OS and unfavorable characteristics regarding differentiation degree and stage in patients with ESCC.^[6]^ In our study, the preoperative NLR level was significantly associated with survival in both univariate and multivariate analysis (Table 2). Recently, PA has become a hot protein due to its short half-life, close association with the nutrient status and prognosis of survival.^[19]^ In addition to Alb, PA can also be used as a reliable index to value the nutritional status of patients. Qiang Zhao et al showed that the NLR/Alb ratio was an independent prognostic factor for patients with ESCC.^[9]^ This study also showed that there was negative correlations between Alb and NLR. Alb was significantly associated with OS only in univariate analysis, but not in multivariate analysis.^[9]^ This similar result was also can be found in our study (Table 2). Pre-treatment NLR/Alb remained independent predictor of pathological complete response in patients with rectal cancer following neoadjuvant chemoradiation.^[20]^ One study have showed that serum Alb or NLR was an independent risk factor for the survival of patients of EC. And the AUC of PA was 0.644 and the AUC of Alb was 0.543 in this study, therefore, PA may have a more sensitive value for predicting survival of patients than Alb.^[19]^ Our study also showed the index of PA had a higher AUC area than that of the index of Alb, but the difference is not statistically significant (*P* > .05, Fig. 3 and Table 3). However, the index of NLR/ PA had a higher AUC area than that of the index NLR/Alb (*P* = .032, Table 3). The reason may be that the half-life of PA (nearly 2 days) is shorter than Alb (nearly 20 days).^[19]^ However, there is no research on the correlation between the NLR/PA and EC prognosis. Additionally, whether the index of NLR/PA ratio is better than the NLR/Alb in survival prognosis remains unknown. Our study showed that there was a negative correlation between PA and NLR. The AUC area of NLR/PA was significantly higher than that of NLR or NLR/Alb (*P* = .032, Table 3). But the AUC area of PA was not significantly higher than the areas of NLR or Alb (Table 3). In subgroup analysis, we found the AUC area of NLR/PA was significantly higher than the areas of NLR or NLR/Alb only in stage I-II. In stage III-IVA, the index of NLR was superior to the index of NLR/PA or NLR/Alb (Fig. 4). The mechanism remains unclear. A meta-analysis showed elevated NLR was associated with stage in patients with ESCC. Patients with advanced stages had higher NLR than that of patients with early stage.^[6]^ This may be explain that NLR is a best prognostic indicator for patients with stage III-IVA.

Inflammation and nutritional status plays a critical role in cancer progression and prognosis. However, the mechanism remains unclear. Patients with newly diagnosed EC usually have impaired nutrition status and higher inflammatory status.^[11]^ One study found the level of vascular endothelial growth factor (VEGF) was inversely correlated with the levels of Alb, PA, and NLR. And VEGF has an important role in the progression of malignant neoplasms.^[21]^ Albumin can not only reflect the body's nutritional status, but also can be used as an index of the body's inflammation status.^[22]^ Albumin plays an important role in cancer in a variety of ways, including through direct inhibition of growth and induction of tumor cell apoptosis.^[23]^ Long-term inflammation causes damage to the vascular endothelium and increases vascular permeability resulting in decreased serum albumin levels.^[22]^ Previous studies have demonstrated that inflammation is associated with PA.^[24]^ The negative correlations between NLR and Alb or PA were also showed in our study. In addition, neutrophils that can inhibit T cells and have been recognized as an important element in tumor progression.^[25]^ In total, malnutrition has weakened the immune system of patients and consequently has induced poor prognosis. Furthermore, inflammation can change the body's internal environment, thus leading to the occurrence and progression of malignancy.^[26]^

This study showed that the preoperative NLR/PA ratio is an independent predictor of patients with ESCC for the first time. The index of NLR/PA can simultaneously reflect the body's immune function and nutritional status. The index of NLR/PA was superior to NLR and NLR/Alb in survival prognosis for patients with early stage (stage I-II) ESCC, with the advantages of economical and easily measurable. However, there are several limitations to our study. First, this study is observational and all data is retrospectively obtained. Second, the number of enrolled cases is relatively small. Moreover, this is a single-center study. Thus, the findings of our study are expected more large-sample trials to confirm in future.

## Author contributions

Jiandong Zhang was responsible for the final decision to submit for publication or others. Yajuan Lv had the full data of the paper and write the report. Zhen Liu and Fengjun Liu helped to gather data. Yuan Tian did the literature search.

**Data curation:** Zhen Liu, Yuan Tian, Fengjun Liu.

**Writing – original draft:** Yajuan Lv.

**Writing – review & editing:** Jiandong Zhang.

Jiandong Zhang orcid: 0000-0003-0169-7092.
